# Quality of life following adult veno-venous extracorporeal membrane oxygenation for acute respiratory distress syndrome: a systematic review

**DOI:** 10.1007/s11136-021-02834-0

**Published:** 2021-04-07

**Authors:** E. R. Kurniawati, V. G. H. Rutjens, N. P. A. Vranken, T. S. R. Delnoij, R. Lorusso, I. C. C. van der Horst, J. G. Maessen, P. W. Weerwind

**Affiliations:** 1 Department of Cardiothoracic Surgery, Maastricht University Medical Center+, P. Debyelaan 25, PO Box 5800, 6202 AZ Maastricht, the Netherlands; 2 Department of Intensive Care Medicine, Maastricht University Medical Center+, Maastricht, the Netherlands; 3 Department of Cardiology, Maastricht University Medical Center+, Maastricht, the Netherlands; 4 Cardiovascular Research Institute Maastricht (CARIM), Maastricht University, Maastricht, the Netherlands

**Keywords:** Veno-venous extracorporeal membrane oxygenation, Veno-venous extracorporeal life support, Adult, Acute respiratory distress syndrome, Health-related quality of life

## Abstract

**Background:**

Veno-venous extracorporeal membrane oxygenation (VV-ECMO) has been used successfully for the past decade in adult patients with acute respiratory distress syndrome (ARDS) refractory to conventional ventilatory support. However, knowledge of the health-related quality of life (HRQoL) in VV-ECMO patients is still limited. Thus, this study aimed to provide a comprehensive overview of the HRQoL following VV-ECMO support in ARDS patients.

**Methods:**

A systematic search was performed on PubMed and Web of Science databases from January 1st, 2009 to October 19th, 2020. Studies reporting on HRQoL following VV-ECMO for ARDS in adults were included. Two authors independently selected studies, extracted data, and assessed methodological quality.

**Results:**

Eight studies were eligible for inclusion, consisting of seven observational studies and one randomized controlled trial (total *N* = 441). All eight studies had a quantitative design and reported 265 VV-ECMO survivors to have a reduced HRQoL compared to a generally healthy population. Follow-up time varied between six months to three years. Additionally, only four studies (total *N* = 335) compared the HRQoL of VV-ECMO (*N* = 159) to conventionally treated survivors (*N* = 176), with one study showing a significantly better HRQoL in VV-ECMO survivors, while three studies were stating comparable HRQoL across groups. Notably, most survivors in these studies appeared to experience varying degrees of anxiety, depression, and post-traumatic stress disorder (PTSD).

**Conclusions:**

ARDS survivors supported by VV-ECMO have a decline in HRQoL and suffered from physical and psychological impairments. This HRQoL reduction is comparable or even better to the HRQoL in conventionally treated ARDS survivors.

## Introduction

Acute respiratory distress syndrome (ARDS) is a frequent cause of respiratory failure in critical care patients. It is defined by the acute onset of non-cardiogenic pulmonary edema and hypoxemia, which might require mechanical ventilation [1]. While the ARDS incidence covers 10% of all ICU admissions, 25% of these patients have severe ARDS leading to profound hypoxemia [2]. There are limited therapeutic options for ARDS patients [3, 4], mainly based on conventional mechanical ventilation and supportive care. Despite recent technological advances in ventilatory support, mortality rates remain high in this patient population (27–45%) [5].

Veno-venous extracorporeal membrane oxygenation (VV-ECMO) has been successfully employed in adult patients with severe ARDS refractory to conventional ventilatory support [6, 7]. The use of ECMO as an adjunct to lung-protective ventilation strategies has been suggested to ameliorate ventilator-induced and ventilator-associated lung injury [8]. The Extracorporeal Life Support Organization (ELSO) registry showed that the number of adults treated with VV-ECMO doubled during the H1N1 global pandemic from 200 cases in 2008 to 495 cases in 2009 [9]. Moreover, ECMO has also been applied in severe respiratory compromised patients suffering from the on-going global coronavirus disease (COVID-19) caused by the novel severe acute respiratory syndrome coronavirus-2 (SARS-CoV-2) [10, 11]. While still little is known on the true efficacy of ECMO in the COVID-19 setting, the natural resemblance of COVID-19 and seasonal influenza’s complications with respect to acute onset and symptoms prompt to ECMO implantation in most severe pulmonary decompensated patients [12, 13]. Despite the increased VV-ECMO application in the last decade, survival rates barely improved, with a current survival rate ranging from 56 to 64% [14]. Besides clinical endpoints such as survival and survival time, only a few studies to date focused on outcomes in terms of quality of life in patients receiving VV-ECMO for ARDS.

Health-related quality of life (HRQoL) is a multidimensional construct that describes the perceived impact of health status, including physical, psychological, and social domains of health [15]. The results from a former review on adult VV-ECMO survivors indicate varying degrees of a reduced HRQoL [16]. Moreover, there is evidence that ARDS survivors may experience physical impairment and psychiatric symptoms following ICU discharge [17–19]. Zwischenberger and Pitcher stated that patients often require thorough assistance and rehabilitation, including physical, occupational, nutritional, and speech therapy after successful weaning from ECMO support [20]. The cognitive, psychiatric, and physical impairments have shown to recover between 6 and 12 months following ICU discharge [21], while in some cases, physical issues can prevail for over 3 years [22]. These morbidities contribute to a significant reduction in HRQoL following ECMO. To date, merely a few studies focused on the HRQoL in ARDS patients treated with VV-ECMO [23, 24] and the majority of the available studies did not discriminate between VV-ECMO and veno-arterial ECMO (VA-ECMO) patients [25–27]. While VV-ECMO provides solely pulmonary support, the VA-ECMO configuration provides both cardiac and pulmonary support, these parameters affect the indication and possibly the HRQoL following therapy [22]. Notably, the median duration of VA-ECMO support is shorter (median of 4 days) [28] compared to VV-ECMO support (median of 10 days) [29]. For these reasons, HRQoL may show different outcomes in VA-ECMO and VV-ECMO survivors. Given the increase of VV-ECMO applications to support refractory gas exchange in ARDS patients [30] and specifically, COVID-19 ARDS-related patients, a better understanding of HRQoL in these patients is warranted.

Despite the increasing number of reports describing the HRQoL of patients treated with ECMO, a study focusing specifically on HRQoL in ARDS patients supported by VV-ECMO is still lacking. Additionally, actual HRQoL scores are not always described or displayed in previous studies, which makes interpretation of the effect of VV-ECMO therapy on HRQoL in ARDS-related patients challenging. The present systematic review aims to describe HRQoL and long-term outcomes in adult ARDS patients supported by VV-ECMO.

## Methods

### Literature search strategy

**Fig. 1:**
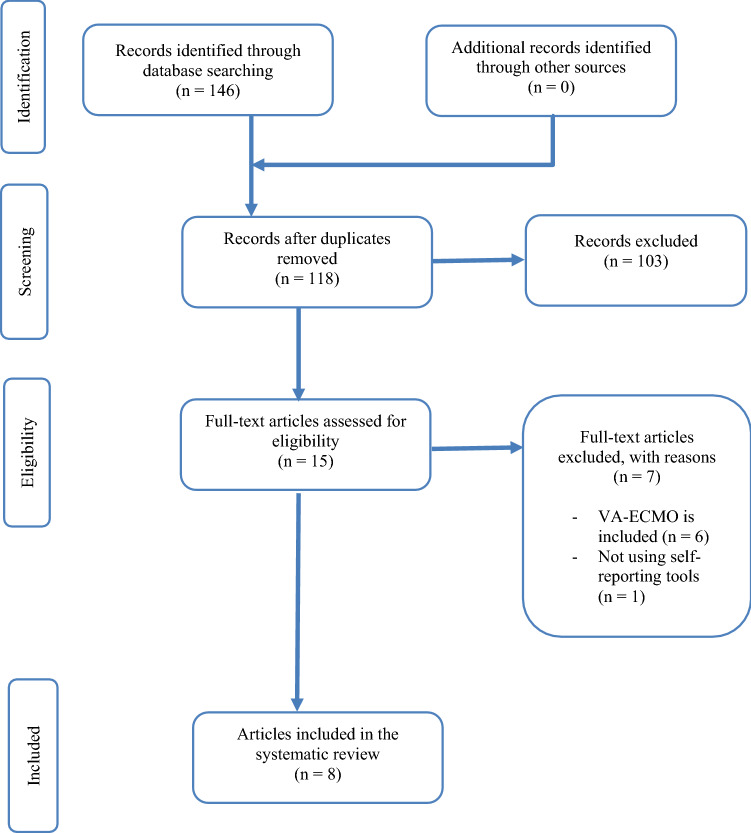
Flowchart of the search strategy

A systematic search was performed independently by two reviewers (EK and VR) utilizing the PubMed and Web of Science databases and was completed on October 19th, 2020. This search combined Medical Subject Headings (MeSH) and free search terms. The MeSH and free search terms related to VV-ECMO, HRQoL, and ARDS were used to optimize the database search output. The search string was computed as follows: “Extracorporeal Membrane Oxygenation” OR “ECMO” OR “Extracorporeal Life Support” OR “ECLS” OR “VV-ECMO” OR “VV-ECLS” OR “venovenous ECMO” OR “venovenous ECLS” AND “quality of life” OR QoL” OR “SF-36” OR “EuroQoL” OR “EQ-5D” AND “disability” OR “physical disability” OR “health problem” OR “emotional problem” OR “social problem” OR “general health” OR “long-term outcome”. The search was conducted in PubMed and Web of Science databases were conducted separately using the same MeSH and free terms. Search results were combined and reviewed to omit duplicate papers. Acquired articles were checked for relevancy step by step, as depicted in Fig. 1. The Preferred Reporting Items for Systematic Review and Meta-Analysis (PRISMA) [31] guidelines were used for reporting the results.

### Inclusion and exclusion criteria

The Population, Intervention, Comparison, Outcome and Study Design (PICOS) approach was used for the selection of studies included in the systematic search (Table 1). Studies reporting on HRQoL following VV-ECMO in adult patients with ARDS were included. The HRQoL comprises an individual’s perceived health as self-reported physical, mental, and social functioning [15, 32]. Thus, an approach that integrates the individual’s definition of his or her perceived “good” quality of life is likely to be the best indicator of subjective HRQoL [33, 34]. Furthermore, articles that did not assess HRQoL using the most commonly used self-reporting tools such as Short Form-36 (SF-36) and EuroQol-5 dimension (EQ-5D) [15, 35] were excluded in the current review. Articles that did not differentiate between VV-ECMO and VA-ECMO patients, review articles, conference and paper abstracts, editorials, letters, and expert opinions were excluded. Additionally, only full-text articles written in English and published between January 1st, 2009 and October 19th, 2020 were reviewed.

**Table 1:** “Population, intervention, comparison, outcome and study design” (PICOS) approach for the selection of studies following systematic search

Population	Adult patients with acute respiratory distress syndrome (ARDS)
Intervention	VV-ECMO
Comparison	Comparison with those treated with mechanical ventilation
Outcome	Health-related quality of life
Study design	Prospective and retrospective cohort studies Randomized controlled trial

### Study selection

Two reviewers (EK and VR) independently assessed all studies for inclusion and extracted potentially relevant studies. The eligibility of the articles was determined by screening and reviewing the full-text article. Studies that did not answer the current research question were eliminated. Any potential disagreements regarding eligibility were resolved by consensus among three members of the research team (EK, VR, and PW). Agreement on study inclusion was examined using Cohen’s kappa coefficient to assess inter-rater reliability [36]. Next, information including the first author’s last name, publication year, country of origin of the study, study characteristics, and the HRQoL study results were retrieved.

### Assessment of risk of bias in included studies

Two researchers (EK and VR) performed the risk of bias assessment independently. Based on the study design, the Quality Assessment Tool for Observational Cohort and Cross-Sectional Studies from the National Heart, Lung and Blood Institute [37] and the Cochrane Risk of Bias Tool [38] were used to assess the study quality. Risk of bias was evaluated in all included studies following major criteria: risk of selection bias, precision, risk of information bias, adequate assessment of the association between exposure and outcome, and risk of investigator bias. Any discrepancies between the researchers were discussed until reaching a consensus or involving a third researcher (PW). Agreement between the two researchers was analyzed using Cohen’s kappa test [36].

## Results

### Study selection

The initial search from PubMed and Web of Science databases yielded a total of 146 studies. Duplicate studies were removed after which 118 studies remained eligible. The initial screening of titles and abstracts excluded all studies that did not evaluate the HRQoL of ARDS patients supported with VV-ECMO. Studies that did not evaluate HRQoL using self-reporting tools were also removed. Two researchers (EK and VR) reviewed the remaining 15 studies for full manuscript review. As a result, seven studies were excluded because they did not specifically evaluate the HRQoL of VV-ECMO (*n* = 6) or did not use self-reporting tools to evaluate HRQoL (*n* = 1). Ultimately, a total of eight studies [39–46] were reviewed, as depicted in Fig. 1. There was almost perfect agreement on study inclusion between the two researchers, *κ* = 0.87 (95% CI 0.62–1.12), *p* = 0.001.

### Characteristics of the studies

From the total of eight included studies, seven were of observational nature [39–42, 44–46] and one was a randomized control trial [43]. Four of the seven included quantitative studies were retrospective [39, 41, 42, 45], and the other three were prospective and observational studies [40, 44, 46]. Due to the observational design, randomization or blinding was not performed in most studies. Three studies originated from Italy [39, 40, 44], one from Australia [41], one from Ireland [42], one from the UK [43], one from France [45], and another from China [46]. Six studies concerned a single-center design [39, 41, 42, 44–46] and two were multi-center studies [40, 43]. Four studies [39, 41, 42, 44] were only examining VV-ECMO survivors, while the other four studies [40, 43, 45, 46] also included ARDS patients undergoing conventional treatment.

The total number of surviving ARDS patients treated with VV-ECMO or conventional ventilatory support was 441, i.e., 265 in the VV-ECMO group [39–46] and 176 patients in the conventional management group (total from four studies [40, 43, 45, 46]). The population of patients in both groups consisted of predominantly male patients (on average 62.8%), except for the study by Hodgson et al. which concerned a relatively small proportion of male patients (48%) [41]. Overall, the age ranged from 36 to 54 years in both groups, with VV-ECMO patients being slightly younger than the conventional management group. Across all included studies, the median ICU stay was between 11 and 46 days, and follow-up was conducted between 6 and 32 months following hospital discharge. Other study characteristics are summarized in Table 2.

**Table 2:** Study characteristics

Parameter	First author and origin of the study							
	Galazzi et al. [39] Italy	Grasselli et al. [40] Italy	Hodgson et al. [41] Australia	O’Brien et al. [42] Ireland	Peek et al. [43] UK	Sanfilippo et al. [44] Italy	Sylvestre et al. [45] France	Wang et al. [46] China
Study design	Quantitative retrospective observational study (cohort)	Quantitative prospective observational study (cohort)	Quantitative retrospective observational study (cohort)	Quantitative retrospective observational study (cohort)	Quantitative randomized controlled trial (RCT)	Quantitative prospective observational study (cohort)	Quantitative retrospective observational study (cohort)	Quantitative prospective observational study (cohort)
Total population (N)—HRQoL sample size								
VV-ECMO	26–17	26–18	18–15	19–13	57–52	43–33	49–22	27–24
MV	N/A	31–19	N/A	N/A	46–32	N/A	36–18	63–48
Sex (M) (%)								
VV-ECMO	12 (70%)	24 (70%)	10 (48%)	7 (54%)	51 (57%)	24 (73%)	12 (55%)	18 (75%)
MV	N/A	31 (62%)	N/A	N/A	53 (59%)	N/A	11 (61%)	33 (69%)
Age (years)								
VV-ECMO	49 (38–55)	54 (41–63)	36.3 ± 12.1	44 ± 11	39.9 ± 13.4	45.0 ± 9.8	41 (32–56)	38.0 ± 15.1
MV	N/A	54 (45–70)	N/A	N/A	40.4 ± 13.4	N/A	51 (43–63)	44.3 ± 15.6
VV-ECMO duration (days)								
VV-ECMO	19 (15–33)	9 (6–13)	10.6 (3.6–15.8)	15 (11–19)	9 (6–16)	10 (7–15)	12 (8–19)	6.0 ± 2.3
MV	N/A	N/A	N/A	N/A	N/A	N/A	N/A	N/A
MV duration (days)								
VV-ECMO	36 (12–74)	21 (11–35)	15.3 (12.0–23.2)	3 (2–20) of pre-ECMO	10 (4.8–22.8)	2 (1–4) days pre-ECMO and 9 (4–16) days post-ECMO	36 (28–64)	10.0 (6.0–16.3)
MV	N/A	8 (5–21)	N/A	N/A	11 (4.0–20.3)	N/A	29 (21–46)	9.0 (6.0–13.0)
ICU stay (days)								
VV-ECMO	37 (20–79)	24 (15–36)	20.7 (14.9–28.6)	31 (25–74)	24 (13.0–40.5)	2 (1–7) but only pre-ECMO	46 (34–71)	13.0 (9.8–22.3)
MV	N/A	11 (5–25)	N/A	N/A	13 (11–16)	N/A	35 (24–47)	11.0 (8.0–18.0)
Hospital stay (days)								
VV-EMCO	N/A	33 (19–48)	28.4 (18.5–37.7)	N/A	35 (15.6–74.0)	4 (2–8) but only pre-ECMO	61 (45–99)	25.5 (16.5–31.3)
MV	N/A	23 (12–45)	N/A	N/A	17 (4.8–45.3)	N/A	55 (43–90)	26.0 (15.0–56.3)
Discharge destination								
VV-ECMO	N/A	92% to home; 8% to other hospital	44% to home; 50% to other hospital; 6% to rehabilitation facility	47% to referral hospital; 23% to a rehabilitation center; 31% to home	N/A	N/A	N/A	92% to home; 8% to other hospital
MV	N/A	90% to home; 10% to other	N/A	N/A	N/A	N/A	N/A	90% to home; 10% to other
Follow-up time (months)								
VV-ECMO	17 (14–25)	12	8.4 (6–16)	36 (14–39)	6	2.7 (2–5) years	20 (17–22)	12.7 ± 5.8
MV	N/A	12	N/A	N/A	6	N/A	22 (18–23)	14.8 ± 6.5
HRQoL assessment tool(s)	EQ-5D	SF-36, SGRQ, IES-R	SF-36, EQ-5D	SF-36, HADS, IES-R	SF-36, EQ-5D, SGRQ, HADS, mini-mental examination, and specific questions about sleep	SF-36, HADS, CES-D, IES-R	SF-36, BDI-IA, BAI, IES	SF-36, EQ-5D
Reported HRQoL outcomes	Perceived QoL = 75%; 60% showed good outcomes physically and psychosocially; 71% returned to their normal working activities	One-year survival was similar between VV-ECMO and MV; Both groups had almost full recovery of lung function; MV patients reported more fatigue, weakness, and limitation in daily activities; VV-ECMO survivors had higher HRQoL scores and lower PTSD rates than non-ECMO survivors	Mean VV-ECMO SF-36 scores were significantly lower compared to matched healthy controls for all domains except bodily pain and role-emotional; 42% of survivors were unable to perform usual activities and described severe or extreme anxiety and depression; 80% of survivors had no problem with personal care; 52% returned to work and 29% returned to previous work level at the follow-up time	Significantly lower mean SF-36 of physical functioning and physical component scores for VV-ECMO survivors. No statistical difference was found in the SF-36 scores between VV-ECMO survivors and matched general population in Ireland for the following domains: mental health, social function, vitality, and a mental component. Results from the HADS questionnaire showed that seven participants (54%) of VV-ECMO survivors experienced anxiety of which five of whom (38.5%) suffered from severe anxiety. Two participants (15%) showed a HADS-D score of ≥ 8, which is associated with depression. Three participants (23%) were considered to be at risk for PTSD. Four of the six (67%) of participants had returned to work at the follow-up time	No significant differences were found between VV-ECMO and MV for EQ-5D, SF-36, St George’s hospital respiratory questionnaire, hospital anxiety and depression scale, and mini-mental state examination	Physical role limitations and general-health perceptions were the worst SF-36 domains (25 and 56, respectively). Psychological tests showed high risk of depression (39–42%, patients; 39–52%, caregivers), anxiety (42% patients; 39% caregivers), and PTSD (47% patients; 61% caregivers). Patients depression or anxiety scores were correlated to age and the outcome reported by caregivers	Lower cognitive function was experienced by 55% of ECMO and 56% of MV survivors. ECMO and MV survivors had similar depressive symptoms (36% vs 39%, respectively), as well as anxiety symptoms (55% vs 44%, respectively) and PTSD (33% vs 44%, respectively). At the follow-up time, 46% of VV-ECMO had returned to their original work as compared to 67% of MV survivors	No statistically differences were found in EQ-between VV-ECMO and MV scores; 13% VV-ECMO and 15% MV reported fatigue and decreased endurance; 42% VV-ECMO and 27% MV reported symptoms of anxiety or depression; 67% VV-ECMO and 50% MV survivors returned to work

VV-ECMO = veno-venous extracorporeal life support, MV = mechanical ventilation (considered to be the conventional treatment group); EQ-5D = EuroQol 5-Dimensions; SF-36 = Short Form 36; SGRQ = St. George’s Respiratory Questionnaire; IES-R = Impact of Event Scale-Revised; HADS = Hospital Anxiety and Depression Scale; N/A = not available; CES-D = Centre for Epidemiologic Studies Depression; BDI-IA = Beck Depression Inventory; BAI = Beck Anxiety Inventory; Values are expressed as mean ± standard deviation or median (25% IQR-75% IQR)

### HRQoL of the studies

#### HRQoL evaluation tools

Based on the inclusion criterion, all included studies used either SF-36 or EQ-5D, or both. Additionally, some studies also used other HRQoL evaluation tools as a combination, such as St. George’s Respiratory Questionnaire (SGRQ), Impact of Event Scale-Revised Score (IES-R), Hospital Anxiety and Depression Scale (HADS), mini-mental state examination, Centre for Epidemiologic Studies Depression (CES-D), the shortened Beck Depression Inventory, and the Beck Anxiety Inventory.

#### HRQoL results of ARDS patients following VV-ECMO

Regardless of the variety of follow-up time, all included studies showed a decrement in the HRQoL score of ARDS patients following VV-ECMO. Mean SF-36 scores were significantly lower for VV-ECMO survivors compared to the matched general population. Mobility problems were reported by VV-ECMO survivors in two studies [39, 41]. Additionally, VV-ECMO survivors reported a varying degree (15–54%) of mental health symptoms, i.e., anxiety, depression, and PTSD [41, 42, 44–46]. Despite the reduced HRQoL, over half of the ARDS patients treated with VV-ECMO returned to work during the follow-up time [39, 41, 46].

#### HRQoL of ARDS patients treated with VV-ECMO vs. conventional ventilatory support

The majority of the studies reported similar HRQoL between ARDS patients treated with VV-ECMO and conventional ventilatory support. Only one study reported better HRQoL at the follow-up time for VV-ECMO patients compared to survivors treated conventionally [40]. Signs of anxiety, depression, and PTSD were reported in both treatment groups. Survivors of VV-ECMO showed to have a higher incidence of anxiety and depression (range 36–55%) than those treated conventionally (range 27–44%) [45, 46]. Conversely, more patients in the conventional treatment group (44%) suffered from PTSD compared to the patients in the VV-ECMO group (33%) [45]. Only two studies reported a return-to-work rate, one reported a lower return to work rate for VV-ECMO (46%) than for survivors treated conventionally (67%) [45], while the other study reported conversely (67% for VV-ECMO and 50% for conventional respiratory support) [46]. Additionally, a similar incidence of fatigue and decreased endurance were reported by both groups (13% for VV-ECMO and 15% for conventional respiratory support) [46].

### Risk of bias within studies

Two researchers independently performed the risk of bias assessment using the Quality Assessment Tool for Observational Cohort and Cross-sectional studies from the National Heart, Lung and Blood Institute [37] and The Cochrane Risk of Bias Tool for randomized controlled trials [38]. The Quality Assessment Tool for Observational Cohort and Cross-sectional studies [37] and The Cochrane Risk of Bias Tool for randomized controlled trials [38] consist of 14 and 7 items, respectively. Both tools can be applied using three categories: low risk of bias, high risk of bias, or unclear risk of bias. The researchers compared the assessment results; discrepancies were discussed and resolved by agreement. The overview of the risk of bias assessment is depicted in Tables 3 and 4. Seven of the included studies [39–42, 44–46] were assessed with The Quality Assessment Tool for Observational Cohort and Cross-sectional studies from the National Heart, Lung and Blood Institute. The study by Peek et al. [43] appeared to have a low risk of bias after assessment by the Cochrane Risk of Bias Tool. On the risk of bias assessment, the agreement between the two researchers was almost perfect, *κ* = 0.94 (95% CI 0.86–1.02), *p* < 0.001. Assessment of risk of bias across studies was not performed.

**Table 3:** Assessment of bias using the quality assessment tool for observational cohort and cross-sectional studies from the National Heart, Lung and Blood Institute

	Galazzi et al. [39]	Grasselli et al. [40]	Hodgson et al. [41]	O’Brien et al. [42]	Sanfilippo et al. [44]	Sylvestre et al. [45]	Wang et al. [46]
1. Was the research question or objective in this paper clearly stated?	Y	Y	Y	Y	Y	Y	Y
2. Was the study population clearly specified and defined?	Y	Y	Y	N	Y	Y	Y
3. Was the participation rate of eligible persons at least 50%?	Y	Y	Y	Y	Y	N	Y
4. Were all the subjects selected or recruited from the same or similar populations (including the same time period)?	Y	Y	Y	Y	Y	Y	Y
Were inclusion and exclusion criteria for being in the study prespecified and applied uniformly to all participants?	Y	Y	Y	Y	Y	Y	Y
5. Was a sample size justification, power description, or variance and effect estimates provided?	N	N	N	N	N	N	N
6. For the analyses in this paper, were the exposure(s) of interest measured prior to the outcome(s) being measured?	N	Y	N	Y	Y	Y	Y
7. Was the timeframe sufficient so that one could reasonably expect to see an association between exposure and outcome if it existed?	Y	Y	Y	Y	Y	Y	Y
8. For exposures that can vary in amount or level, did the study examine different levels of the exposure as related to the outcome (e.g., categories of exposure, or exposure measured as continuous variable)?	N/A	N/A	N/A	N/A	N/A	N/A	N/A
9. Were the exposure measures (independent variables) clearly defined, valid, reliable, and implemented consistently across all study participants?	Y	Y	Y	Y	Y	Y	Y
10. Was the exposure(s) assessed more than once over time?	N	N	N	N	N	N	N
11. Were the outcome measures (dependent variables) clearly defined, valid, reliable, and implemented consistently across all study participants?	Y	Y	Y	Y	Y	Y	Y
12. Were the outcome assessors blinded to the exposure status of participants?	N/A	N/A	N/A	N/A	N/A	N/A	N/A
13. Was loss to follow-up after baseline 20% or less?	Y	Y	Y	Y	Y	Y	Y
14. Were key potential confounding variables measured and adjusted statistically for their impact on the relationship between exposure(s) and outcome(s)?	N/A	N/A	N/A	N/A	N/A	N/A	N/A

*Y* yes, *N* no, *N/A* not applicable

**Table 4:** Assessment of bias using the cochrane risk of bias tool for randomized controlled trials for Peek et al. [43]

Domain	Source of bias	Support for judgement	Review authors’ judgement
Selection bias	Random sequence generation	“Patients were enrolled from three types of centres: the ECMO centre at Glenfield Hospital, Leicester, which treated all patients who were randomly allocated for consideration to receive ECMO; tertiary intensive care units (conventional treatment centres); and referral hospitals, which sent patients to the conventional treatment centres if they were randomly allocated to receive continued conventional management.”	Low risk
	Allocation concealment	“Patients were randomly allocated by minimisation in a 1:1 ratio to conventional management by intermittent positive-pressure ventilation or high-frequency oscillatory ventilation, or both, or consideration for treatment by ECMO. Minimisation factors were type of centre; age; hours of high-pressure or high FiO_2_ ventilation; presence of hypoxia or hypercarbia; diagnostic group; and number of organs failed.”	High risk
Performance bias	Blinding of participants and personnel	Blinding of participants and personnel was not described	Unclear risk
	Blinding of outcome assessment	Blinding of outcome assessment was not described	Unclear risk
Attrition bias	Incomplete outcome data	“Consequently, the number of patients with missing data are lower than for other components of EQ-5D, and other follow-up and economic assessments.”	High risk
Reporting bias	Selective reporting	The primary and secondary outcomes are identifiable in the published report	Low risk
Other bias	Other source bias	None were identified	Low risk

## Discussion

Given the increased use of VV-ECMO to support refractory gas exchange in ARDS patients, efforts should be devoted to gain a better understanding of the HRQoL in ECMO survivors to ultimately improve patient care following ECMO support. While earlier systematic reviews lack focus specifically on the HRQoL of ARDS patients supported by VV-ECMO, the current review assessed the HRQoL of adult VV-ECMO survivors, indicating a lower HRQoL in these patients compared to the general healthy population.

Eight studies were included in this review, which revealed that VV-ECMO survivors have lower SF-36 scores, i.e., reduced physical, mental, and social dimension scores compared to the general healthy population norms. This is consistent with previous studies showing reduced SF-36 scores in most SF-36 domains [24, 47]. Nevertheless, these results should be interpreted with caution, as they may be attributable to other factors than treatment using VV-ECMO by itself, for example, the length of hospital stay or severity of the underlying disease [48]. Although some information regarding the HRQoL in ECMO survivors is available, data revealing the VV-ECMO survivors’ experience related to their health problems occurring after discharge from the hospital remains scarce. To explore the problems and health needs of patients who had been successfully weaned from VV-ECMO, a qualitative study is necessary to provide a better understanding of the patient’s experiences [49, 50]. Insight into the patient’s health needs and physical, psychological, and social problems that occur after discharge from the hospital will contribute to the initiation of additional treatment modalities. Previous studies emphasize the importance of qualitative studies to provide an invaluable perspective of the patients’ needs across all quality of life domains [51, 52].

Notably, as compared to patients supported conventionally, selected studies suggested no reduction of HRQoL for VV-ECMO survivors [40, 43, 45, 46]. One study reported a better HRQoL in VV-ECMO survivors [40]. In contrast, three others reported similar HRQoL across the two groups—although patients treated with VV-ECMO had more severe underlying pathology compared to those treated conventionally [43, 45, 46]. Wang and colleagues argued that the difference in respiratory support mode might explain the comparable HRQoL between VV-ECMO and conventionally treated survivors. The ventilatory strategies used in VV-ECMO patients might have offered better protection against lung injury resulting in improved long-term outcomes [46].

Similarly, Peek et al. suggested that the comparable HRQoL outcomes between both groups might result from the fact that VV-ECMO protects the pulmonary system from high pressure and FiO_2_ ventilation, allowing a minimum iatrogenic contribution to lung injury [43]. Additionally, the clinics included in this study were highly experienced centers; hence, optimal outcomes were to be expected [43]. On the other hand, Grasselli et al. reported improved HRQoL, as shown by a lower median reduction of SF-36 scores in VV-ECMO survivors compared to their non-ECMO counterparts [40]. The authors did not find a clear explanation of why VV-ECMO survivors showed better long-term outcomes in just the general health domain. They hypothesized that an improved HRQoL in VV-ECMO survivors might be attributable to ultra-protective ventilation during ECMO support, which may have reduced the risk of polyneuropathy and myopathies associated with mechanical ventilation [40]. This may explain the improved outcome in VV-ECMO patients, despite a longer mechanical ventilation duration in this population. Additionally, advanced care provided by medical and paramedical professionals, psychological support, and resource teams received by VV-ECMO patients during their hospital stay (i.e., nutrition, wound care, physical therapy) should also be considered when comparing HRQoL [40]. On the other hand, given the non-interventional nature of the study, patient selection bias may have occurred, as shown by the reduced number of comorbidities in ECMO patients [40].

Hodgson et al. [41] reported that 52% of the VV-ECMO survivors had returned to work eight months after hospital discharge, and 26% managed to return to their previous working levels. Other studies showed higher return-to-work rates ranged between 67 and 71% at least a year post-hospital discharge [39, 42, 46]. Interestingly, despite longer support times, patients receiving active and passive physiotherapy in awake VV-ECMO [39], showed a similar return-to-work rate as the patients with shorter support times [42, 46]. Meanwhile, the results from Sylvestre et al. [45] showed that although their follow-up was longer (2 years after discharge), their observed return to work rate was considerably lower (46%) than the study by Hodgson et al. (52%) [41] and Wang et al. (67%) [46]. They argued that their patient cohort was older than the patients in the studies by Hodgson et al. [41] and Wang et al. [46].

Galazzi et al. reported that early rehabilitation should be strived for ICU patients, especially for ECMO patients [40], to minimize ICU-related weakness and fatigue [53]. Therefore, despite the more extended ICU stay, an acceptable degree of autonomy was achieved post-treatment in their study in an effort to improve general outcomes [39]. Additionally, the follow-up was performed in a later phase than in the study conducted by Wang et al. [46] (17 vs. 12.7 months), and patients, therefore, had more time to recover before follow-up took place. According to Schmidt et al. [24], a longer follow-up significantly improved SF-36 scores in role physical and role emotional domains in VV-ECMO survivors. Notably, ARDS survivors discharged from the ICU following conventional treatment showed to have a lower (50%) return-to-work rate compared to VV-ECMO survivors (67%)[46].

In several studies, the prevalence of physical impairment in VV-ECMO survivors was higher than the prevalence of mental impairment at various follow-up points between 12 months and 3 years after discharge [42, 44, 46]. Although the factors contributing to long-term physical impairment remain unclear, a ubiquitous ICU-acquired weakness and potential ECMO-specific sequelae may play a significant role [54, 55]. Reduced mobility, pain, or discomfort in the legs or feet was reported as VV-ECMO survivors’ main issues contributing to physical impairment [40, 41]. This can be explained by the fact that prolonged cannulation of the femoral veins can trigger localized nerve ischemia resulting in paraesthesia and limited mobility of the lower limbs [55].

It is well known that ICU survivors treated for ARDS, exposed to life-threatening circumstances, are prone to suffer from PTSD [47, 56]. This is confirmed by several previous studies that reported that a majority of ARDS survivors treated with VV-ECMO or conventional ventilatory support are suffering from anxiety and depression or PTSD [39, 43, 44, 57]. Both groups showed decreased HRQoL, especially on the role physical in VV-ECMO and emotional domain in conventionally treated survivors [40, 46]. However, survivors treated conventionally displayed a higher risk of PTSD [40, 43, 45, 47]. Additionally, these survivors appeared to have more limitations regarding physical activities, such as fatigue, weakness, restricted daily activities, and psychological issues interfering with their former way of life due to PTSD [40]. These observations have led the way for other investigators to evaluate neurocognitive dysfunction and its risk factors in ARDS patients supported by ECMO [45]. One challenge for studies considering long-term outcomes is knowing the baseline HRQoL status of the patients admitted due to ARDS. Thus, the extent to which the HRQoL deficits manifested due to ARDS is not always clear. Moreover, the deficits may be a function of prolonged and severe critical illness rather than specifically from ARDS or ECMO.

### Limitations

Several limitations should be noted in this review. Of the eight included studies, seven were of observational design and thereby lacked in randomization and blinding. Due to this study’s inclusion criteria, such as language and HRQoL reporting instruments, potentially valuable articles may not have been included. More importantly, since the included studies utilized self-reporting instruments, outcomes were highly subjective, implying heterogeneity in HRQoL outcomes, and thereby comparison of results and making specific conclusions and recommendations is hampered. The included studies offer valuable yet highly heterogeneous data, as there is variability in, e.g., reporting instruments and follow-up time. Additionally, most of the included studies were conducted in a single-center, had relatively small sample sizes, and thus may have lacked the power to adequately detect possible group differences. All included studies had a quantitative observational purpose, which does not capture detailed descriptions of patients’ experiences during and after VV-ECMO support [58]. Lastly, although the included studies’ HRQoL was encouraging, the true magnitude of long-term impairments may be biased by survival.

## Conclusion

The present systematic review describes a reduced HRQoL in ARDS survivors supported by VV-ECMO and suggests this reduction to be similar to observations in conventionally treated ARDS survivors. Based on the quantitative design of the included studies and to gain further insight into the quality of life of VV-ECMO survivors, additional qualitative studies are warranted.

## Abbreviations

ARDS: Acute respiratory distress syndrome
CES-D: Centre for epidemiologic studies depression
COVID-19: Coronavirus disease 2019
ECMO: Extracorporeal membrane oxygenation
ELSO: Extracorporeal life support organization
EQ-5D: EuroQol-5 dimension
HADS: Hospital anxiety and depression scale
HRQoL: Health-related quality of life
H1N1: Hemagglutinin 1 neurominidase 1 (influenza A)
ICU: Intensive care unit
IES-R: Impact of event scale-revised score
MeSH: Medical subject headings
MV: Mechanical ventilation
PRISMA: Preferred reporting items for systematic review and meta-analysis
PTSD: Post-traumatic stress disorder
QoL: Quality of life
SARS-CoV-2: Severe acute respiratory syndrome coronavirus-2
SF-36: Short form-36
SGRQ: St. George’s respiratory questionnaire
VA-ECMO: Veno-arterial extracorporeal membrane oxygenation
VV-ECMO: Veno-venous extracorporeal membrane oxygenation
